# High systemic immune inflammation index values are associated with prolonged length of hospital stay in patients with acute exacerbation of chronic obstructive pulmonary disease: a retrospective cohort study

**DOI:** 10.3389/fmed.2025.1711893

**Published:** 2026-01-13

**Authors:** Jiming Xiao, Yunqiu Liu, Liying Zheng, Qing Yang, Xinxin Hao, Yibo Zhao, Dongmei Chen, Baojing Feng, Liye Wang

**Affiliations:** 1 Kailuan General Hospital Affiliated to North China University of Science and Technology, Tangshan, China; 2 Department of Pulmonary and Critical Care Medicine, Kailuan General Hospital, Tangshan, China

**Keywords:** acute exacerbation, chronic obstructive pulmonary disease, prolonged length of hospital stay, retrospective cohort study, Systemic Immune Inflammation Index

## Abstract

**Objectives:**

This study aimed to investigate the association between the Systemic Immune Inflammation Index (SII) and the prolonged length of hospital stay (PLOS) in patients with acute exacerbation of chronic obstructive pulmonary disease (AECOPD).

**Methods:**

A retrospective analysis was conducted involving 986 patients aged ≥ 40 years with AECOPD admitted to Kailuan General Hospital between January 2018 and December 2024. PLOS was defined as a stay exceeding 7 days. Complete blood counts were collected within 24 h of admission to calculate the SII, which was the log-transformed and denoted as In-SII. Logistic regression analysis was employed to compare the predictive value of In-SII and In-NLR (neutrophil-to-lymphocyte ratio) for PLOS in patients with AECOPD. Additionally, restricted cubic splines (RCS) and decision curves analysis (DCA) were utilized to explore the nonlinear relationship and clinical net benefit between In-SII and PLOS in patients with AECOPD.

**Results:**

In-SII was an independent risk factor for PLOS in patients with AECOPD (odds ratios for Model 1, Model 2, and Model 3 were 1.527, 1.294, and 1.496, respectively; *p* < 0.05). Its predictive performance is superior to In-NLR. According to RCS curves, there was a linear association between In-SII and PLOS in patients with AECOPD (Model 1: *p* for nonlinear = 0.664; Model 2: *p* for nonlinear = 0.663; Model 3: *p* for nonlinear = 0.571). Additionally, DCA indicated a significant net clinical benefit when the In-SII threshold ranged from 0.41 to 0.80.

**Conclusion:**

High SII serves as an independent risk factor for PLOS in patients with AECOPD. This indicated that patients with AECOPD exhibiting high SII levels have poorer outcomes, necessitating earlier implementation of more robust intervention measures and close monitoring of disease progression.

## Introduction

Chronic obstructive pulmonary disease (COPD) is a common chronic respiratory disorder characterized by persistent airflow limitation, posing a significant threat to global health (1). As the third leading cause of death worldwide, COPD accounts for approximately three million deaths annually (2). Acute exacerbations of COPD (AECOPD) are common events in the disease course, with about half of COPD patients experiencing at least one exacerbation per year (3, 4). AECOPD not only accelerates disease progression and reduces quality of life but also imposes substantial economic burdens on patients and healthcare systems (5). Such episodes typically require medical intervention for resolution, placing immense strain on already limited healthcare resources (6).

Length of hospital stay (LOS) serves as a key indicator for assessing resource consumption and economic burden in AECOPD patients, while also closely correlating with disease severity and clinical prognosis (7, 8). Prolonged LOS (PLOS) translates to higher medical costs and poorer clinical outcomes (9). Consequently, identifying biomarkers capable of effectively predicting PLOS is critically important, holding significant value for early intervention and improving patient prognosis. Currently, traditional inflammatory markers such as C-reactive protein (CRP) and Leukocyte count, along with novel inflammatory indicators like the neutrophil-to-lymphocyte ratio (NLR) and platelet-to-lymphocyte ratio (PLR), have been employed to assess AECOPD prognosis (10–13). However, their predictive value remains limited.

The Systemic Immune Inflammation Index (SII) is a novel peripheral blood biomarker integrating platelet count, neutrophil count, and lymphocyte count to reflect both local immune responses and systemic inflammatory states. This index was first proposed by Hu et al. in 2014 (14). Since then, SII has been extensively validated as a robust prognostic indicator in gastric cancer, non-small cell lung cancer, and urothelial carcinoma (15–17). However, relevant research in the field of AECOPD remains relatively limited. Therefore, this study aims to explore the association between SII and PLOS through a retrospective analysis of 986 AECOPD patients, providing evidence for early risk identification and optimization of treatment strategies.

## Materials and methods

### Study participants

This retrospective study included hospitalized patients with AECOPD treated at Kailuan General Hospital from January 2018 to December 2024. The study was approved by the Medical Ethics Committee of the Kailuan General Hospital (No. 2024033).

The Inclusion Criteria included the following: (1) Patients met the diagnostic criteria for AECOPD as defined by the “Expert Consensus on the Acute Exacerbation of Chronic Obstructive Pulmonary Disease in China (revision in 2023)”; (2) Age ≥40 years; (3) For patients with repeated hospitalizations, the most recent admission record was selected. Exclusion Criteria: (1) Incomplete medical records; (2) Patients who died during hospitalization; (3) Patients with acute infections outside the lungs; and (4) Patients with malignancies, hematological disorders, or immune system diseases.

### Data collection

This study retrieved demographics, comorbidities, medical history, peripheral biomarkers, lung function test, and treatment interventions. The demographics included age, gender, LOS, Body Mass Index (BMI), smoking, and drinking history. The comorbidities included hypertension, diabetes, coronary heart disease, cerebrovascular disease, and pneumonia. The medical history included hospitalization due to AECOPD in the prior year. The peripheral biomarkers included leukocyte count, neutrophil count, monocyte count, lymphocyte count, eosinophil count, basophil count, erythrocyte count, hemoglobin count, platelet count, albumin, creatinine, and CRP, were all collected within 24 h of patient admission. The lung function test included GOLD categories. The treatment interventions included intravenous corticotherapy during hospitalization, antibiotics during hospitalization, the need for theophylline, diuretics, and oxygen therapy.

The SII was calculated using the following formula: SII = (platelet count × neutrophil count)/lymphocyte count.

The primary outcome was LOS, based on preliminary research findings (average hospitalization duration for AECOPD patients is 7 days) (18–21). Patients were divided into two groups for comparative analysis: the normal LOS (NLOS) group (LOS ≤ 7 days) and the PLOS group (LOS > 7 days).

### Statistical analysis

SPSS 22.0 and the R language were used for data analysis. The original SII values failed the normality test (Kolmogorov–Smirnov test) and were found to be non-normally distributed (*p* < 0.05). To satisfy the normality assumption for parametric tests, data underwent a log transformation (base *e*). Non-normally distributed continuous variables were described using median and interquartile range, and compared between groups using the Mann–Whitney *U*-test. Categorical variables were described using counts and percentages and compared between groups using chi-square tests. The variance inflation factor (VIF) test was used to test the collinearity of variables. Construct univariate and multivariate logistic regression models to evaluate the predictive ability of In-SII and In-NLR for PLOS with AECOPD. A restricted cubic spline (RCS) was used to explore the correlation between ln-SII and PLOS in AECOPD patients. The decision curve analysis (DCA) was used to examine the predictive value of ln-SII for PLOS. *p* < 0.05 is considered to be statistically significant.

## Results

The information on patients with AECOPD is shown in Table 1. A total of 986 patients were divided into the NLOS group 379 (38.4%) and the PLOS group 607 (61.6%). The two groups differed in hypertension, hospitalization due to AECOPD in the prior year, leukocyte count, neutrophil count, monocyte count, lymphocyte count, albumin, CRP, In-SII, GOLD categories, intravenous corticotherapy during hospitalization, antibiotics during hospitalization, diuretics, and oxygen therapy. Patients in the PLOS group had a higher prevalence of hypertension and a greater proportion of hospitalization due to AECOPD in the prior year ≥1 time (*p* < 0.05). For peripheral biomarkers, elevated levels of leukocyte count, neutrophil count, monocyte count, albumin, CRP, and In-SII, but lower lymphocyte count levels were observed in the PLOS group (all *p* < 0.05). In addition, the PLOS group accounted for a higher proportion of GOLD categories in 3–4, intravenous corticotherapy during hospitalization, antibiotics during hospitalization, diuretics, and oxygen therapy. However, there was no difference in age, gender, BMI, smoking and drinking history, diabetes, coronary heart disease, cerebrovascular disease and pneumonia, eosinophil count, basophil count, erythrocyte count, hemoglobin, platelet count, creatinine, need for theophylline (all *p* > 0.05).

**Table 1:** Comparison of main characteristics between the NLOS group and the PLOS group in AECOPD patients.

**Variable**	**NLOS group**	**PLOS group**	***P***
	***N*** = **379**	***N*** = **607**	
**Demographics**			
Age, years	66.0 (59.0, 71.0)	67.0 (60.0, 71.0)	0.174
**Gender**, ***n*** **(%)**			0.522
Male	297 (78.4%)	465 (76.6%)	
Female	82 (21.6%)	142 (23.4%)	
BMI (kg/m^2^)	24.22 (21.60, 26.57)	24.22 (21.78, 26.83)	0.583
Smoking history, *n* (%)	251 (66.2%)	385 (63.4%)	0.371
Drinking history, *n* (%)	116 (30.6%)	167 (27.5%)	0.296
**Comorbidities**, ***n*** **(%)**			0.020
Hypertension	171 (45.1%)	320 (52.7%)	
Diabetes	54 (14.2%)	109 (18.0%)	0.127
Coronary heart disease	101 (26.6%)	197 (32.5%)	0.053
Cerebrovascular disease	95 (25.1%)	187 (30.8%)	0.052
Pneumonia	211 (55.7%)	362 (59.6%)	0.220
**Medical history**			
**Hospitalization due to AECOPD in the prior year**, ***n*** **(%)**			0.001
≥1	43 (11.3%)	117 (19.3%)	
**Peripheral biomarkers**			
Leukocyte count, 10^9^/L	6.3 (5.2, 7.5)	6.6 (5.4, 8.1)	0.026
Neutrophil count, 10^9^/L	3.7 (3.0, 4.8)	4.1 (3.2, 5.3)	<0.001
Monocyte count, 10^9^/L	0.42 (0.34, 0.53)	0.44 (0.35, 0.56)	0.037
Lymphocyte count, 10^9^/L	1.7 (1.4, 2.2)	1.6 (1.2, 2.1)	0.004
Eosinophil count, 10^9^/L	0.15 (0.07, 0.29)	0.14 (0.07, 0.26)	0.422
Basophil count, 10^9^/L	0.03 (0.02, 0.05)	0.03 (0.02, 0.05)	0.473
Erythrocyte count, 10^9^/L	4.48 (4.11, 4.84)	4.44 (4.05, 4.77)	0.113
Hemoglobin, g/L	138 (128, 148)	136 (125, 147)	0.115
Platelet count, 10^9^/L	217 (179, 258)	221 (180, 269)	0.188
Albumin, g/L	41 (38, 43)	40 (37, 42)	0.001
Creatinine, μmol/L	65 (55, 74)	67 (57, 77)	0.098
**C-reactive protein**, ***n*** **(%)**			<0.001
>6 mg/L	116 (30.6%)	259 (42.7%)	
In-SII	6.104 (5.773, 6.593)	6.311 (5.920, 6.769)	<0.001
**Lung function test**			
**GOLD categories**, ***n*** **(%)**			0.015
GOLD1-2	260 (68.6%)	370 (61.0%)	
GOLD3-4	119 (31.4%)	237 (39.0%)	
**Treatment interventions**			
**Intravenous corticotherapy during hospitalization**, ***n*** **(%)**			<0.001
Yes	21 (5.5%)	92 (15.2%)	
**Antibiotics during hospitalization**, ***n*** **(%)**			0.001
Yes	307 (81.0%)	538 (88.6%)	
**Need for theophylline**, ***n*** **(%)**			0.305
Yes	282 (74.4%)	469 (77.3%)	
**Diuretics**, ***n*** **(%)**			0.029
Yes	28 (7.4%)	71 (11.7%)	0.029
**Oxygen therapy**, ***n*** **(%)**			<0.001
Yes	108 (28.5%)	258 (42.5%)	

NLOS Group, normal length of hospital stay group; PLOS Group, prolonged length of hospital stay group; AECOPD, acute exacerbation of chronic obstructive pulmonary disease; BMI, body mass index; FEV1, forced expiratory volume in 1 s; SII, Systemic Immune-Inflammatory Index; GOLD, the Global Initiative for Chronic Obstructive Lung Disease.

Since neutrophil count, lymphocyte count, and platelet count were included in the calculation of SII, the three variables were excluded from subsequent analyses to avoid collinearity. We further tested the collinearity of the significant baseline variables. With a VIF value >2 as a threshold, Leukocyte (VIF = 2.226) was eliminated, and the variables (hypertension, hospitalization due to AECOPD in the prior year, monocyte count, albumin, CRP, In-SII, GOLD categories, intravenous corticotherapy during hospitalization, antibiotics during hospitalization, diuretics, oxygen therapy) remained. As shown in Table 2.

**Table 2:** Collinearity analysis.

**Variable**	**Variance inflation factor**
Hypertension	1.042
Hospitalization due to AECOPD in the prior year	1.024
Leukocyte count	2.226
Monocyte count	1.681
Albumin	1.108
*C*-reactive protein	1.270
In-SII	1.710
GOLD categories	1.102
Intravenous corticotherapy during hospitalization	1.100
Antibiotics during hospitalization	1.128
Diuretics	1.070
Oxygen therapy	1.231

AECOPD, acute exacerbation of chronic obstructive pulmonary disease; SII, Systemic Immune-Inflammatory Index; GOLD, the Global Initiative for Chronic Obstructive Lung Disease.

Comparative predictive value of In-SII and In-NLR for the risk of PLOS in Patients with AECOPD. As shown in Table 3, in the univariate model (Model 1), both In-SII and In-NLR were significantly associated with PLOS (OR = 1.527 and OR = 1.576, respectively, both *p* < 0.05). After adjusting for confounders with VIF < 2 (Model 2), In-SII remained significantly independent (OR = 1.294, *p* = 0.018), while the association of In-NLR became non-significant (OR = 1.356, *p* = 0.073). This preliminary finding suggests that SII may possess more robust predictive capability than NLR when accounting for the same confounders. To directly validate this, Model 3 included both In-SII and In-NLR. Results showed In-SII retained significant predictive value (OR = 1.496, *p* = 0.008), whereas In-NLR was no longer significant. Subgroup analyses across three groups yielded consistent conclusions. In summary, SII is not only an independent predictor of PLOS but also demonstrates superior predictive value compared to NLR.

**Table 3:** Comparative predictive value of In-SII and In-NLR for the risk of PLOS in patients with AECOPD: based on continuous and categorical variables.

**Variable**	**Model 1**		**Model 2**		**Model 3**	
	**OR(95%CI)**	***P***	**OR(95%CI)**	***P***	**OR(95%CI)**	***P***
In-SII	1.527 (1.262, 1.848)	<0.001	1.294 (1.045, 1.602)	0.018	1.496 (1.109, 2.019)	0.008
In-NLR	1.576 (1.269, 1.958)	<0.001	1.356 (1.074, 1.712)	0.073	0.832 (0.496, 1.398)	0.488
**In-SII tertiles**						
(3.542, 5.980)	Ref.		Ref.		Ref.	
(5.981, 6.531)	1.664 (1.217, 2.275)	0.001	1.558 (1.126, 2.156)	0.007	1.630 (1.088, 2.443)	0.018
(6.535, 9.260)	1.959 (1.426, 2.690)	<0.001	1.445 (1.014, 2.059)	0.042	1.383 (0.802, 2.386)	0.043
**In-NLR tertiles**						
(−1.040, 0.620)	Ref.		Ref.		Ref.	
(0.630, 1.070)	1.324 (0.970, 1.808)	0.077	1.166 (0.842, 1.616)	0.355	0.888 (0.592,1.335)	0.569
(1.080, 3.930)	1.890 (1.375, 2.597)	<0.001	1.360 (0.958, 1.931)	0.086	1.057 (0.615, 1.818)	0.841

Model 1 without adjustment; Model 2 adjusting Hypertension, Hospitalization due to AECOPD in the prior year, Monocyte count, Albumin, C-reactive protein, GOLD categories, Intravenous corticotherapy during hospitalization, Antibiotics during hospitalization, Diuretics, Oxgen therapy ; Model 3 adjusting Model 2 + In-NLR + In-SII (when continuous variable) or Model 2 + In-NLR tertiles or In-SII tertiles (when categorical variables).

In-NLR, In (Neutrophil count divided by Lymphocyte count).

SII, Systemic Immune-Inflammatory Index; NLR, neutrophil-to-lymphocyte ratio; PLOS, prolonged length of hospital stay; AECOPD, acute exacerbation of chronic obstructive pulmonary disease; GOLD, the Global Initiative for Chronic Obstructive Lung Disease; OR (95% CI), odds ratio (95% confidence interval).

**Figure 1:**
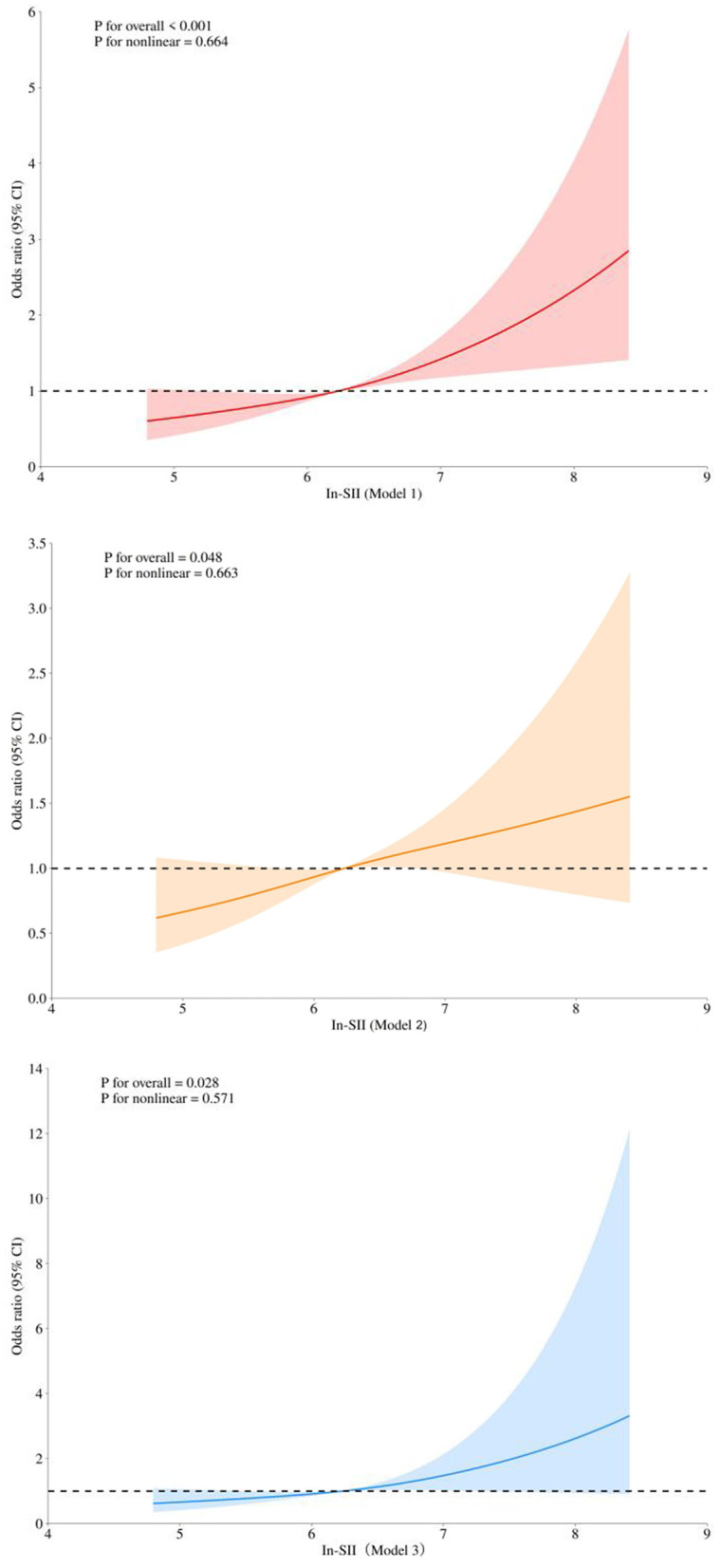
Restricted cubic splines of the association between the In-SII and the risk of PLOS in patients with AECOPD. Model 1 without adjustment; Model 2 adjusting Hypertension, Hospitalization due to AECOPD in the prior year, Monocyte count, Albumin, C-reactive protein, GOLD categories, Intravenous corticotherapy during hospitalization, Antibiotics during hospitalization, Diuretics, Oxgen therapy ; Model 3 adjusting Model 2 + In-NLR + In-SII. SII, Systemic Immune-Inflammatory Index; PLOS, prolonged length of hospital stay; AECOPD, acute exacerbation of chronic obstructive pulmonary disease; GOLD, the Global Initiative for Chronic Obstructive Lung Disease; OR (95% CI), odds ratio (95% confidence interval).

Figure 1 displays three RCS regression models. In the following three models, a significant linear correlation exists between In-SII and the risk of PLOS in AECOPD patients (Model 1: overall *p* < 0.001, *p* for nonlinear = 0.664; Model 2: overall *p* = 0.048, *p* for nonlinear = 0.663; Model 3: overall *p* = 0.028, *p* for nonlinear = 0.571).

**Figure 2:**
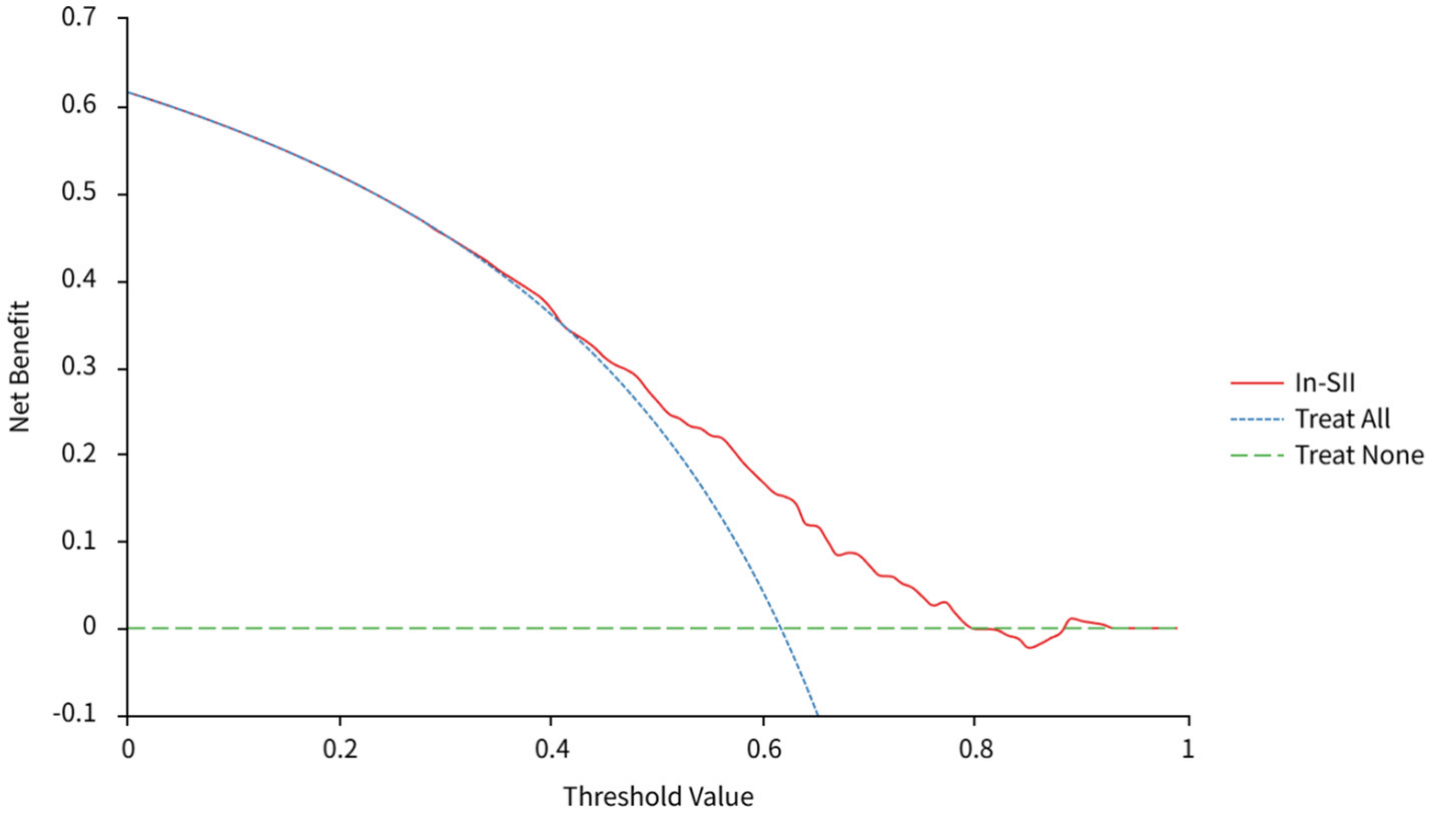
The predictive value of ln-SII for PLOS in patients with AECOPD. SII, Systemic Immune-Inflammatory Index; PLOS, prolonged length of hospital stay; AECOPD, acute exacerbation of chronic obstructive pulmonary disease.

As shown in Figure 2. In order to study the clinical value of In-SII, we explored its predictive value by DCA analysis. When the threshold was about 0.41–0.80, the clinical net benefit of ln-SII was higher than the treat none model and the treat all model. These suggested that ln-SII had good clinical value.

## Discussion

This study is a single-center retrospective investigation that collected laboratory indicators of In-SII to determine its association with PLOS in patients with AECOPD. Multivariate logistic regression analysis revealed that In-SII is an independent risk factor for predicting PLOS in patients with AECOPD, with significantly superior predictive performance compared to In-NLR. This association remained statistically significant even after adjusting for other potential confounding factors. These findings suggest clinicians may utilize SII as a prognostic indicator for AECOPD patients, aiding in the early identification of those at risk for PLOS and enabling enhanced intervention strategies.

Inflammation is a key mechanism in the pathogenesis of AECOPD (1), often triggered by exogenous stimuli such as viral infections, bacterial infections, and air pollution (22). These stimuli lead to massive activation of inflammatory cells within the airways, releasing multiple inflammatory mediators that collectively contribute to and exacerbate persistent damage to airway and alveolar structures (23). This results in significant airflow limitation and airway hyper responsiveness (23). Studies have shown that 70% of COPD patients exhibit elevated levels of at least one inflammatory marker (24). Serum CRP, NLR, fibrinogen, and TNF-α have been extensively studied as inflammatory biomarkers for AECOPD (25, 26). However, the search for more objective, scientifically validated, and readily accessible inflammatory markers, such as the SII derived from routine blood tests, has gained prominence. Recent research has revealed an association between elevated SII levels and increased COPD incidence (27), as well as a higher risk of all-cause mortality among COPD patients with elevated SII levels (28).

This study found that In-SII serves as an independent and superior biomarker to In-NLR for predicting PLOS in patients with AECOPD. NLR primarily reflects the imbalance between neutrophils and lymphocytes. In-SII, however, further integrates the critical dimension of platelets. Elevated SII, characterized by increased platelet and neutrophil counts accompanied by decreased lymphocyte counts, suggests a proinflammatory hematologic state. The potential mechanisms linking this condition to PLOS may be as follows: activated neutrophils release neutrophil extracellular traps (NETs) (29), which inevitably exacerbate lung tissue damage during their functional activity (30). Concurrently, the release of substantial proinflammatory mediators intensifies systemic inflammation (31), leading to PLOS. Second, lymphocytes contribute to tissue injury by secreting inflammatory cytokines, including IL-4, IL-5, and IFN-γ (32). However, persistent inflammation itself induces increased lymphocyte apoptosis and reduced numbers, diminishing pathogen clearance capacity and increasing susceptibility to secondary infections (33), thereby significantly PLOS. Finally, platelets play crucial roles not only in hemostasis and thrombosis but also in inflammatory and immune responses (34, 35). During AECOPD, activated platelets interact with neutrophils, monocytes, and endothelial cells to form microthrombi. This impairs microcirculation, causing tissue ischemia and hypoxia, ultimately damaging organ function (36). Therefore, as an emerging composite indicator, SII can capture the complex pathological network leading to PLOS more comprehensively than NLR. Inflammation also contributes to numerous complications, including infections, sepsis, and organ failure, all of which contribute to PLOS.

Currently, there is no unified criterion for determining the PLOS in patients with AECOPD. Different studies have employed varying standards (9, 37, 38). Given that Mushlin's (39) research indicated approximately 90% of AECOPD patients no longer experience complications or require close monitoring after 6 days of hospitalization, they recommended a necessary hospitalization duration of 6–9 days based on patient clinical characteristics. Additionally, Crisafulli et al. (9) found that LOS >7 days correlates with disease severity. Therefore, this study selected 7 days as the threshold for PLOS in AECOPD patients.

This study has several limitations. First, although we collected data from 986 patients, the single-center retrospective design may not reflect broader populations. Second, this study analyzed only the SII values at admission, lacking dynamic monitoring of SII during hospitalization. This limitation restricted our ability to assess the association between the evolution of the inflammatory response and clinical outcomes. Future prospective multicenter cohort studies should be conducted to further validate the prognostic value of SII. Continuous monitoring of SII dynamics during hospitalization should be pursued to develop a more precise risk prediction model based on inflammatory kinetics, thereby guiding clinical treatment decisions.

## Conclusion

High SII levels were independently associated with a higher risk of PLOS in patients with AECOPD. Clinicians should closely monitor elevated SII values and initiate more aggressive anti-inflammatory or antimicrobial treatment regimens at an early stage to reduce the risk of prolonged hospitalization. This approach improves patient outcomes, enhances quality of life, and conserves healthcare resources.

## Data Availability

The raw data supporting the conclusions of this article will be made available by the authors, without undue reservation.

## References

[1] Venkatesan P. GOLD COPD report: 2024 update. Lancet Respir Med. (2024) 12:15–6. doi: 10.1016/S2213-2600(23)00461-7 38061380

[2] Safiri S Carson-Chahhoud K Noori M Nejadghaderi SA Sullman MJM Ahmadian Heris J . Burden of chronic obstructive pulmonary disease and its attributable risk factors in 204 countries and territories, 1990–2019: results from the Global Burden of Disease Study 2019. BMJ. (2022) 378:e069679. doi: 10.1136/bmj-2021-069679 35896191 PMC9326843

[3] Kim V Aaron SD. What is a COPD exacerbation? Current definitions, pitfalls, challenges and opportunities for improvement. Eur Respir J. (2018) 52:1801261. doi: 10.1183/13993003.01261-2018 30237306

[4] Butler CC Gillespie D White P Bates J Lowe R Thomas-Jones E . C-Reactive protein testing to guide antibiotic prescribing for COPD exacerbations. N Engl J Med. (2019) 381:111–20. doi: 10.1056/NEJMoa1803185 31291514

[5] Soler-Cataluna JJ. Severe acute exacerbations and mortality in patients with chronic obstructive pulmonary disease. Thorax. (2005) 60:925–31. doi: 10.1136/thx.2005.040527 16055622 PMC1747235

[6] Jeyachandran V Hurst JR. Advances in chronic obstructive pulmonary disease: management of exacerbations. Br J Hosp Med. (2022) 83:1–7. doi: 10.12968/hmed.2022.0275 35938767

[7] Li M Wang F Chen R Liang Z Zhou Y Yang Y . Factors contributing to hospitalization costs for patients with COPD in China: a retrospective analysis of medical record data. COPD. (2018) 13:3349–57. doi: 10.2147/COPD.S175143 30349238 PMC6190824

[8] Dang-Tan T Zhang S Tavares RV Stutz M Ismaila AS Vaillancourt J . The Burden of illness related to chronic obstructive pulmonary disease exacerbations in Québec, Canada. Can Respir J. (2017) 2017:1–10. doi: 10.1155/2017/8184915 28713217 PMC5496115

[9] Crisafulli E Ielpo A Barbeta E Ceccato A Huerta A Gabarrús A . Clinical variables predicting the risk of a hospital stay for longer than 7 days in patients with severe acute exacerbations of chronic obstructive pulmonary disease: a prospective study. Respir Res. (2018) 19:261. doi: 10.1186/s12931-018-0951-4 30591055 PMC6307152

[10] Ellingsen J Janson C Bröms K Hårdstedt M Högman M Lisspers K . Fibrinogen, white blood cells, and blood cell indices as prognostic biomarkers of future COPD exacerbation frequency: the TIE cohort study. JCM. (2024) 13:3855. doi: 10.3390/jcm13133855 38999421 PMC11242174

[11] Fermont JM Masconi KL Jensen MT Ferrari R Di Lorenzo VAP Marott JM . Biomarkers and clinical outcomes in COPD: a systematic review and meta-analysis. Thorax. (2019) 74:439–46. doi: 10.1136/thoraxjnl-2018-211855 30617161 PMC6484697

[12] Zinellu A Zinellu E Mangoni AA Pau MC Carru C Pirina P . Clinical significance of the neutrophil-to-lymphocyte ratio and platelet-to-lymphocyte ratio in acute exacerbations of COPD: present and future. Eur Respir Rev. (2022) 31:220095. doi: 10.1183/16000617.0095-2022 36323421 PMC9724880

[13] Liao QQ Mo YJ Zhu KW Gao F Huang B Chen P . Platelet-to-lymphocyte ratio (PLR), neutrophil-to-lymphocyte ratio (NLR), monocyte-to-lymphocyte ratio (MLR), and eosinophil-to-lymphocyte ratio (ELR) as biomarkers in patients with Acute Exacerbation of Chronic Obstructive Pulmonary Disease (AECOPD). Int J Chron Obstruct Pulmon Dis. (2024) 19:501–18. doi: 10.2147/COPD.S447519 38414718 PMC10898603

[14] Hu B Yang XR Xu Y Sun YF Sun C Guo W . Systemic immune-inflammation index predicts prognosis of patients after curative resection for hepatocellular carcinoma. Clin Cancer Res. (2014) 20:6212–22. doi: 10.1158/1078-0432.CCR-14-0442 25271081

[15] Tong Y-S Tan J Zhou X-L Song Y-Q Song Y-J. Systemic immune-inflammation index predicting chemoradiation resistance and poor outcome in patients with stage III non-small cell lung cancer. J Transl Med. (2017) 15:221. doi: 10.1186/s12967-017-1326-1 29089030 PMC5664920

[16] Chien TM Li CC Lu Y-M Chou YH Chang HW Wu WJ. The predictive value of systemic immune-inflammation index on bladder recurrence on upper tract urothelial carcinoma outcomes after radical nephroureterectomy. J Clin Med. (2021) 10:5273. doi: 10.3390/jcm10225273 34830555 PMC8623909

[17] He K Si L Pan X Sun L Wang Y Lu J . Preoperative systemic immune-inflammation index (SII) as a superior predictor of long-term survival outcome in patients with stage I–II gastric cancer after radical surgery. Front Oncol. (2022) 12:829689. doi: 10.3389/fonc.2022.829689 35296020 PMC8918673

[18] Ruparel M López-Campos JL Castro-Acosta A Hartl S Pozo-Rodriguez F Roberts CM. Understanding variation in length of hospital stay for COPD exacerbation: European COPD audit. ERJ Open Res. (2016) 2:00034–2015. doi: 10.1183/23120541.00034-2015 27730166 PMC5005149

[19] Crisafulli E Torres A Huerta A Guerrero M Gabarrús A Gimeno A . Predicting in-hospital treatment failure (≤7 days) in patients with COPD exacerbation using antibiotics and systemic steroids. COPD. (2016) 13:82–92. doi: 10.3109/15412555.2015.1057276 26451913

[20] Wang H Yang T Yu X Chen Z Ran Y Wang J . Risk factors for length of hospital stay in acute exacerbation chronic obstructive pulmonary disease: a multicenter cross-sectional study. Int J Gen Med. (2022) 15:3447–58. doi: 10.2147/IJGM.S354748 35378912 PMC8976556

[21] Yang L Li M Shu J Yang Y Huang Q. A risk prediction model for prolonged length of stay in patients with acute exacerbations of chronic obstructive pulmonary disease: a retrospective study of 225 patients in a single center in Kunming, China. Med Sci Monit. (2021) 8:e934392. doi: 10.12659/MSM.934392 35136009 PMC8842644

[22] Barnes PJ. Inflammatory endotypes in COPD. Allergy. (2019) 74:1249–56. doi: 10.1111/all.13760 30834543

[23] Hogg JC Timens W. The pathology of chronic obstructive pulmonary disease. Annu Rev Pathol Mech Dis. (2009) 4:435–59. doi: 10.1146/annurev.pathol.4.110807.092145 18954287

[24] Oh JY Sin DD. Lung inflammation in COPD: why does it matter? F1000 Med Rep. (2012) 4:23. doi: 10.3410/M4-23 23236338 PMC3516832

[25] Leshchenko IV Baranova II. Inflammatory biomarkers in chronic obstructive pulmonary disease. Pul′*monologiâ*. (2012) 108–117. doi: 10.18093/0869-0189-2012-0-2-108-117

[26] Gan WQ. Association between chronic obstructive pulmonary disease and systemic inflammation: a systematic review and a meta-analysis. Thorax. (2004) 59:574–80. doi: 10.1136/thx.2003.019588 15223864 PMC1747070

[27] Ye C Yuan L Wu K Shen B Zhu C. Association between systemic immune-inflammation index and chronic obstructive pulmonary disease: a population-based study. BMC Pulm Med. (2023) 23:295. doi: 10.1186/s12890-023-02583-5 37563621 PMC10416535

[28] Benz E Wijnant SRA Trajanoska K Arinze JT De Roos EW De Ridder M . Sarcopenia, systemic immune-inflammation index and all-cause mortality in middle-aged and older people with COPD and asthma: a population-based study. ERJ Open Res. (2022) 8:00628-2021. doi: 10.1183/23120541.00628-2021 35036418 PMC8752940

[29] Qu G Ribeiro HAL Solomon AL Sordo Vieira L Goddard Y Diodati NG . The heme scavenger hemopexin protects against lung injury during aspergillosis by mitigating release of neutrophil extracellular traps. JCI Insight. (2025) 10:e189151. doi: 10.1172/jci.insight.189151 40232861 PMC12128981

[30] Zhang H Wang Y Qu M Li W Wu D Cata JP . Neutrophil, neutrophil extracellular traps and endothelial cell dysfunction in sepsis. Clin Transl Med. (2023) 13:e1170. doi: 10.1002/ctm2.1170 36629024 PMC9832433

[31] Papayannopoulos V Zychlinsky A. NETs: a new strategy for using old weapons. Trends Immunol. (2009) 30:513–21. doi: 10.1016/j.it.2009.07.011 19699684

[32] Barczyk A Pierzchała W Kon OM Cosio B Adcock IM Barnes PJ. Cytokine production by bronchoalveolar lavage T lymphocytes in chronic obstructive pulmonary disease. J Allergy Clin Immunol. (2006) 117:1484–92. doi: 10.1016/j.jaci.2006.02.013 16751017

[33] Cronkite DA Strutt TM. The regulation of inflammation by innate and adaptive lymphocytes. J Immunol Res. (2018) 2018:1–14. doi: 10.1155/2018/1467538 29992170 PMC6016164

[34] Hvas A-M. Platelet function in thrombosis and hemostasis. Semin Thromb Hemost. (2016) 42:183–4. doi: 10.1055/s-0036-1572329 27023375

[35] Morrell CN Aggrey AA Chapman LM Modjeski KL. Emerging roles for platelets as immune and inflammatory cells. Blood. (2014) 123:2759–67. doi: 10.1182/blood-2013-11-462432 24585776 PMC4007605

[36] Zhou Z-P Zhong L Liu Y Yang Z-J Huang J-J Li D-Z . Impact of early heparin therapy on mortality in critically ill patients with sepsis associated acute kidney injury: a retrospective study from the MIMIC-IV database. Front Pharmacol. (2024) 14:1261305. doi: 10.3389/fphar.2023.1261305 38273840 PMC10808568

[37] Wang Y Stavem K Dahl F Humerfelt S Haugen T. Factors associated with a prolonged length of stay after Acute Exacerbation of Chronic Obstructive Pulmonary Disease (AECOPD). Int J Chron Obstruct Pulmon Dis. (2014) 9:99–105. doi: 10.2147/COPD.S51467 24477272 PMC3901775

[38] Harries T Thornton H Crichton S Schofield P Gilkes A White P. Length of stay of COPD hospital admissions between 2006 and 2010: a retrospective longitudinal study. Int J Chron Obstruct Pulmon Dis. (2015) 10:603–11. doi: 10.2147/COPD.S77092 25834419 PMC4370686

[39] Mushlin AI. The necessary length of hospital stay for chronic pulmonary disease. JAMA. (1991) 266:80. doi: 10.1001/jama.1991.03470010084035 1904506

